# Effect of early prophylactic low-dose recombinant human erythropoietin on retinopathy of prematurity in very preterm infants

**DOI:** 10.1186/s12967-020-02562-y

**Published:** 2020-10-19

**Authors:** Huiqing Sun, Juan Song, Wenqing Kang, Yong Wang, Xiantao Sun, Chongchen Zhou, Hong Xiong, Falin Xu, Mingchao Li, Xiaoli Zhang, Zengyuan Yu, Xirui Peng, Bingbing Li, Yiran Xu, Shan Xing, Xiaoyang Wang, Changlian Zhu

**Affiliations:** 1 Department of Neonatology, Children’s Hospital Affiliated to Zhengzhou University, Henan Children’s Hospital, Zhengzhou Children’s Hospital, Zhengzhou, China; 2 Henan Key Laboratory of Child Brain Injury, Institute of Neuroscience and Third Affiliated Hospital, Zhengzhou University, Zhengzhou, 450052 China; 3 Department of Ophthalmology, Children’s Hospital Affiliated to Zhengzhou University, Henan Children’s Hospital, Zhengzhou, China; 4 Key Laboratories of Children’s Genetic Metabolic Diseases, Henan Province, Children’s Hospital Affiliated to Zhengzhou University, Henan Children’s Hospital, Zhengzhou, China; 5 Centre of Perinatal Medicine and Health, Institute of Neuroscience and Physiology, University of Gothenburg, Gothenburg, Sweden; 6 Center for Brain Repair and Rehabilitation, Institute of Neuroscience and Physiology, University of Gothenburg, Gothenburg, Sweden; 7 Department of Women’s and Children’s Health, Karolinska Institutet, Stockholm, Sweden

**Keywords:** Erythropoietin, Retinopathy of prematurity, Preterm infant

## Abstract

**Background:**

Very preterm infants are at risk of developing retinopathy of prematurity (ROP). Recombinant human erythropoietin (rhEPO) is routinely used to prevent anemia in preterm infants; however, the effect of rhEPO on ROP development is still controversial. The purpose of this study was to evaluate the effect of early prophylactic low-dose rhEPO administration on ROP development in very preterm infants.

**Methods:**

A total of 1898 preterm infants born before 32 weeks of gestation were included. Preterm infants received rhEPO (n = 950; 500 U/kg, rhEPO group) or saline (n = 948, control group) intravenously within 72 h of birth and then once every other day for 2 weeks.

**Results:**

The total incidence of ROP was not significantly different between the two groups (10.2% vs*.* 13.2%, p = 0.055). Further analysis showed that rhEPO group had lower rates of type 2 ROP than the control group (2.2% vs*.* 4.1%, RR 0.98; 95% CI 0.96–1.00; *p* = 0.021). Subgroup analysis found that rhEPO treatment significantly decreased the incidence of type 2 ROP in infant boys (1.8% vs. 4.3%, p = 0.021) and in those with a gestational age of 28–29^6/7^ weeks (1.1% vs. 4.9%, p = 0.002) and birth weight of 1000–1499 g (1.2% vs. 4.2%, p = 0.002). There was a small increasing tendency for the incidence of ROP in infants with a gestational age of < 28 weeks after rhEPO treatment.

**Conclusions:**

Repeated low-dose rhEPO administration has no significant influence on the development of ROP; however, it may be effective for type 2 ROP in infant boys or in infants with gestational age > 28 weeks and birth weight > 1500 g.

*Trial registration* The data of this study were retrieved from two clinical studies registered ClinicalTrials.gov (NCT 02036073) on January 14, 2014, https://clinicaltrials.gov/ct2/show/NCT02036073; and (NCT03919500) on April 18, 2019. https://clinicaltrials.gov/ct2/show/NCT03919500.

## Background

Anaemia of prematurity is common in preterm infants, especially in very preterm infants born at < 32 weeks of gestation [1]. It is caused by immaturity of the hematopoietic system, inadequate production of erythropoietin, and iatrogenic blood loss owing to frequent blood sampling [2]. Most very preterm infants receive blood transfusions for the treatment of anaemia during their hospitalisation [3], based on their haemoglobin levels, clinical indicators, and oxygen requirements [4]. Anaemia at birth has also been associated with the development of retinopathy of prematurity (ROP) [5], particularly in preterm infants with a gestational age of < 28 weeks [6]. Moreover, the duration of anaemia during the first week of life is an independent risk factor for ROP, which may be reduced by treating and preventing early anaemia [7]. Previous studies have shown that the early administration of rhEPO is effective in preventing anaemia, thereby reducing the need for red blood cell transfusion in preterm infants [6, 8, 9].

ROP is a proliferative retinal vascular disease affecting the retina of premature infants, and is characterised by neovascularisation, secondary to peripheral retinal ischemia, and subsequent neuro-vascular degeneration [10]. The clinical spectrum of ROP varies from spontaneous regression of the disease to bilateral retinal detachment, which can lead to total blindness [11]. Thus, to reduce the risk of blindness from retinal neovascularisation, retinal vasculopathy should be prevented. rhEPO is a powerful cytoprotective agent that protects both the neurons and vascular cells from apoptosis and mobilises bone marrow progenitor cells into the peripheral bloodstream for vascular repair [12, 13].

However, the effect of rhEPO administration on the development of ROP in preterm infants is still unclear. Vitreous levels of EPO are elevated in preterm infants who developed neovascularisation during phase 2 of ROP [14]. Moreover, serum EPO level was elevated 14 days after birth in preterm infants who developed severe ROP [15, 16], and was reduced 28 days after birth in preterm infants who developed any degree of ROP [17]. Consequently, it remains unclear whether rhEPO treatment is a risk factor for ROP development [1, 18–21]. Thus, we aimed to clarify the effect of rhEPO on ROP development in preterm infants by retrieving data from our previous prospective randomised rhEPO clinical trials in preterm infants (ClinicalTrials.gov: NCT02036073, NCT03919500). The purpose of these clinical trials was to test the hypothesis that early, repeated low-dose administration of rhEPO (500 U/kg) [9, 22], started within 72 h of birth and repeated once every other day for 2 weeks, was safe and would improve the neurological outcomes or reduce the incidence of necrotizing enterocolitis in very preterm infants. In the original clinical trials, ROP screening was routinely performed on premature and low birth weight infants with a gestational age < 32 weeks or birth weight < 2000 g [23]; therefore, the data were available for all preterm infants enrolled in the clinical trials for retrieval of data and reanalysis.

## Methods

### Subjects

Data were retrieved from the original prospective randomised clinical trials performed at the neonatal intensive care units (NICUs) in Zhengzhou University, China. The eligible population for enrolment included infants admitted to the NICU at a gestational age of 24–32 weeks, within 72 h of birth. Infants with any of the following conditions were excluded from the study: genetic or metabolic diseases; major congenital abnormalities; a terminal stage of illness (pH < 7.0 or hypoxia with bradycardia > 2 h); grade III/IV intracranial haemorrhage before randomisation; or lacking parental consent. A block randomisation method stratified by NICU size was used to assign infants 1:1 to either the control or rhEPO group in the previous clinical trials, and block randomisation also stratified by gestational age (< 28 weeks, 28–29^6/7^ weeks, and 30–32 weeks) in NICUs [24]. The study was approved by the Life Science Ethics committee of Zhengzhou University and Henan Medical Academy in accordance with the Declaration of Helsinki.

The original design included several elements to ensure the safety of infants. All study procedures would cease if the infant suffered severe side effects. Criteria for withholding or stopping the study included major venous thrombosis; polycythaemia (haematocrit > 60% or haematocrit increase ≥ 15% not caused by transfusion); and hypertension (systemic blood pressure > 95 mmHg at 0–7 days of age, > 100 mmHg at 8–14 days, or > 105 mmHg at > 14 days) [9]. If the parents wished to withdraw consent at any time, all study procedures would cease.

### rhEPO administration

Eligible infants received 500 U/kg rhEPO intravenously every other day for 2 weeks (a cumulative dose of 3500 U/kg for seven separate intravenous injections, regardless of gestational age; this dose was safe and resulted in improved neurologic outcomes). The first dose was administered within 72 h of birth. Infants in the control group received an equivalent volume of saline, with the same treatment procedure as those in the rhEPO group. Supportive care, including temperature control, parenteral and enteral nutrition, and specific treatment of different clinical problems, were identical between the two treatment groups. Blood transfusion followed the strict clinical criteria [25].

### ROP screening and classification

ROP screening was performed for all very preterm infants (24^0/7^–32) by qualified ophthalmologists with expertise, according to the Chinese guidelines for ROP screening [23]. Follow-up examinations were performed by the examining ophthalmologist based on retinal findings, classified according to the “International classification of retinopathy of prematurity revisited” [26, 27]. ROP was subdivided into stages 1–5 according to the classification and defined as follows [27]: (1) none: immature or mature vascularisation; (2) ROP: stage 1 or stage 2 ROP in zone II or III without plus disease; (3) type 1 ROP: zone I, any stage ROP with plus disease; zone I, stage 3 ROP without plus disease; zone II, stage 2 or 3 ROP with plus disease; (4) type 2 ROP: zone I, stage 1 or 2 ROP without plus disease or zone II, stage 3 ROP without plus disease; and (5) ROP requiring treatment was defined as type 1 ROP or worse. Termination of ROP screening was performed before 45 weeks postmenstrual age (PMA) [26].

### Data collection and study outcomes

Infant and maternal characteristics, delivery information, and outcomes data were collected using a data collection form and entered into a database for subsequent analysis. Study-specific variables, including weekly complete blood cell counts, and the number and volume of blood transfusions during hospitalization, were recorded. Routine blood tests were obtained before and after rhEPO treatment.

This study focused on short-term outcomes before the infant’s 45 weeks of PMA, especially the incidence of ROP (all infants were survival at 45 weeks of PMA). The other short-term outcomes included the incidences of late-onset sepsis defined by a positive blood culture and treatment with antibiotics for ≥ 5 days [28]; bronchopulmonary dysplasia (BPD) defined by persistent parenchymal lung disease, and radiographic confirmation of parenchymal lung disease at 36 weeks PMA in a premature infant [29]; necrotizing enterocolitis (NEC) ≥ stage 2, according to the Bell’s criteria [30]; severe intraventricular hemorrhage (IVH) ≥ grade 3 [31]; and duration of mechanical ventilation.

### Statistical analyses

SPSS software version 21.0 (SPSS Chicago, Illinois, USA) was used for statistical analysis and data management. The outcomes, as well as infant and maternal characteristics, were summarised using descriptive methods and compared using the chi-square test for categorical variables and the t-test for continuous variables. A multivariable logistic regression analysis model was used to adjust for potential confounding factors for type 1 or type 2 ROP. The model was adjusted for birth weight, NEC, and IVH. Interaction analysis in the subgroups was performed using the Mantel–Haenszel tests. The level of statistical significance was two-sided and defined by a p-value < 0.05.

## Results

### Baseline characteristics

**Fig. 1:**
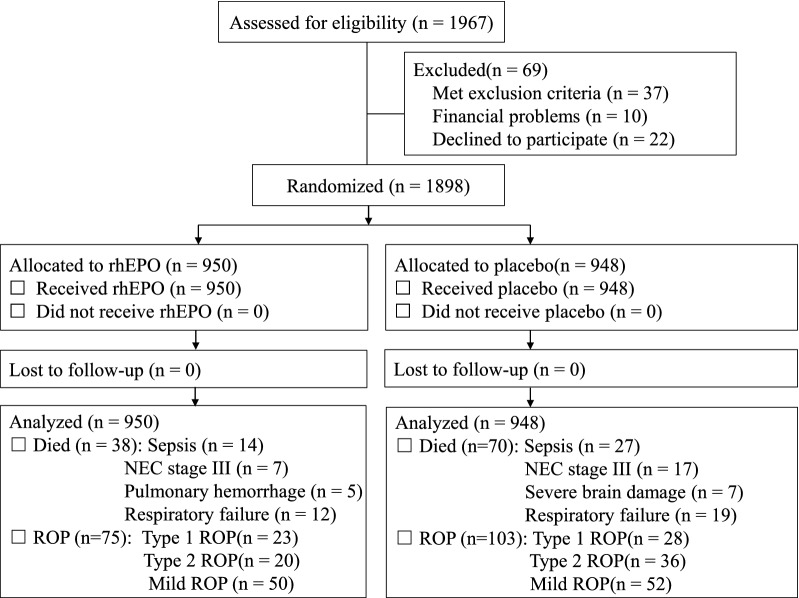
Schematic flowchart shows the numbers of infants who were screened for eligibility, randomly assigned to the rhEPO or control group, and followed up to 45 weeks of corrected age. ROP, retinopathy of prematurity; NEC, necrotizing enterocolitis

A total of 1898 very preterm infants were included (Fig. 1). The baseline characteristics of the infants in the rhEPO and control groups were similar in terms of birth weight and gestational age (Table 1).

**Table 1:** Baseline characteristics of all the infants enrolled in study

Characteristic	Control n = 948	rhEPO n = 950	*P*
Male, n (%)	581 (61.3)	573 (60.3)	0.672
Gestational age (weeks), mean ± SD	29.9 ± 1.5	29.8 ± 1.8	0.413
Birth weight (gram), mean ± SD	1318 ± 252	1307 ± 211	0.298
Maternal age(years), mean ± SD	29.6 ± 5.4	30.0 ± 5.4	0.124
Maternal hypertension, n (%)	199 (21.0)	185 (19.5)	0.424
Premature rupture of membrane, n (%)	255 (26.9)	257 (27.1)	0.959
Twins/multiple births, n (%)	137 (14.5)	110 (11.6)	0.066
Caesarean section, n (%)	403 (42.5)	382 (40.2)	0.328
Surfactant administration, n (%)	410 (43.2)	405 (42.6)	0.817
Clinical chorioamnionitis, n (%)	19 (2.0)	18 (1.9)	0.870
RDS			
Grade 1, n (%)	367 (38.7)	356 (37.5)	0.603
Grade 2, n (%)	79 (8.3)	76 (8.0)	0.802
Grade ≥ 3, n (%)	45 (4.7))	43 (4.5)	0.828
CPAP, n (%)	466 (49.2)	458 (48.2)	0.713
Requirement of oxygen, n (%)	804 (84.8)	780 (82.1)	0.122
Invasive mechanical ventilation, n (%)	327 (34.5)	291 (30.6)	0.078
Early-onset sepsis, n (%)	167 (17.6)	194 (20.4)	0.128

RDS, respiratory distress syndrome; CPAP, continuous positive airway pressure

### Effect of rhEPO on ROP

Overall, there is a tendency of decreased incidence of ROP in the rhEPO group compared to that in the control group; however, the difference between the two groups was not statistically significant (10.2% vs. 13.2%, p = 0.055) (Table 2). The incidence of type 2 ROP was significantly lower in the rhEPO group than in the control group (2.2 vs*.* 4.1%, p = 0.021). The incidence of combined type 1 or 2 ROP was 4.7% in the rhEPO group and 7.3% in the control group (p = 0.022). However, there were no significant differences between the two groups regarding the incidence of type 1 ROP and mild ROP (Table 2). It is important to note that no type of ROP showed a significantly increased incidence in the rhEPO group when compared to the control group (Table 2). There were more infants who developed IVH and NEC in the control group than in the rhEPO group (p = 0.001 and p = 0.012, respectively), and also the mortality rate was higher in the control group (p = 0.001). The rates of late onset sepsis, BPD, and duration of mechanical ventilation were similar between the two groups (Table 2).

**Table 2:** Outcomes of ROP and other outcomes in two groups

Outcomes	Control n = 948	rhEPO n = 950	RR (95% CI)	*P* value
Death, n (%)	70/948 (7.4)	38/950 (4.0)	0.9 (0.94–0.99)	*0.001*
ROP, n (%)	116/878 (13.2)	93/912 (10.2)	0.97 (0.93–1.00)	0.055
Type 1 ROP, n (%)	28/878 (3.2)	23/912 (2.5)	0.99 (0.98–1.01)	0.478
Type 2 ROP, n (%)	36/878 (4.1)	20/912 (2.2)	0.98 (0.96–1.00)	*0.021*
Type 1 or Type 2 ROP, n (%)	64/878 (7.3)	43/912 (4.7)	0.97 (0.95–1.00)	*0.022*
Mild ROP, n (%)	52 /878(5.9)	50/912 (5.5)	1.00 (0.97–1.02)	0.760
Hospital acquired sepsis, n (%)	255 (26.9)	232 (24.4)	0.97 (0.92–1.0)	0.227
BPD, n (%)	210 (22.2)	181 (19.1)	0.96 (0.92–1.01)	0.100
NEC, n (%)	51 (5.4)	29 (3.1)	0.98 (0.96–1.00)	*0.012*
IVH (≥ grade 3), n (%)	72 (7.6)	37 (3.9)	0.96 (0.94–0.98)	*0.001*
Mechanical ventilation, d	3.3 ± 7.4	2.9 ± 5.5	0.41 (-0.39–1.23)	0.313

CI, confidence interval; rhEPO, recombinant human erythropoietin; ROP, retinopathy of prematurity. BPD, bronchopulmonary dysplasia; NEC, necrotizing enterocolitis; IVH, intraventricular hemorrhage

### Subgroup analyses

Subgroup analyses were performed based on gestational age (< 28 weeks, 28–29^6/7^ weeks, 30–32 weeks) [24], birth weight (< 1000 g, 1000–1499 g, ≥ 1500 g), and sex. The incidence of type 1 ROP, type 2 ROP, and combined type 1 or type 2, was found to be associated with gestational age and birth weight (p < 0.01, respectively).

For type 1 ROP, no significant difference was observed in the sex, gestational age, and birth weight subgroup analyses. In infants with a birth weight of < 1,000 g, there was a non-significant increase of type 1 ROP in the rhEPO group (15.9% vs. 9.6%, p = 0.409). However, there were no associations between rhEPO and sex, gestational age, or birth weight for type 1 ROP

**Table 3:** Subgroup interaction analyses in preterm infants with type 1 ROP, type 2 ROP

Type 1 ROP					Type 2 ROP				Type 1 or Type 2 ROP			
Characteristic	Control, n = 28 No. (%)	rhEPO, n = 23 No. (%)	RR (95% CI)	Interaction *P* value	Control, n = 36 No. (%)	rhEPO, n = 20 No. (%)	RR (95% CI)	Interaction *P* value	Control, n = 64 No. (%)	rhEPO, n = 43 No. (%)	RR (95% CI)	Interaction *P* value
Sex				0.391				*0.022*				*0.021*
Boys	19/538 (3.5)	10/549 (1.8)	0.98 (0.96–1.00)		23/538 (4.3)	10/549 (1.8) *	0.98 (0.96–1.00)		42/538 (7.8)	20/549 (3.6) *	0.96 (0.93–0.99)	
Girls	9/340 (2.6)	13/364 (3.6)	1.01 (0.98–1.04)		13/340 (3.8)	10/364 (2.7)	0.99 (0.96–1.02)		22/340 (6.5)	23/364 (6.3)	1.00 (0.96–1.04)	
Gestational age**				0.295				*0.016*				*0.012*
< 28 weeks	6/50 (12.0)	7/65 (10.8)	0.99 (0.86–1.13)		3/50 (6.0)	8/64 (12.5)	1.07 (0.96–1.20)		9/50 (18.0)	15/64 (23.4)	1.07 (0.89–1.30)	
28–29^6/7^ weeks	8/326 (2.5)	10/362 (2.8)	1.00 (0.98–1.03)		16/326 (4.9)	4/362 (1.1) *	0.96 (0.94–0.99)		24/326 (7.4)	14/362 (5.3)	0.96 (0.93–1.00)	
30–32 weeks	14/502 (2.8)	6/485 (1.2)	0.98 (0.97–1.00)		17/502 (3.4)	8/485 (1.6)	0.98 (0.96–1.00)		31/502 (6.2)	14/485 (2.9) *	0.97 (0.94–0.99)	
Birth weight**				0.275				*0.013*				*0.008*
< 1000 g	5/52 (9.6)	10/63 (15.9)	1.07 (0.94–1.24)		7/52 (13.5)	8/63 (12.7)	0.99 (0.86–1.14)		12/52 (23.1)	18/63 (28.6)	1.07 (0.87–1.34)	
1000–1499 g	16/544 (2.9)	12/644 (1.9)	0.99 (0.97–1.01)		23/544(4.2)	8/644 (1.2) *	0.97 (0.95–0.99)		39/544 (7.2)	20/644 (3.1) *	0.96 (0.93–0.98)	
≥ 1,500 g	7/282 (2.5)	1/205 (0.5)	0.98(0.96–1.00)		6/282 (2.1)	4/205 (2.0)	1.00 (0.97–1.02)		13/282 (4.6)	5/205 (2.4)	0.98 (0.95–1.01)	

Probability value is for interaction analysis in subgroups using the Mantel–Haenszel test. p < 0.05 was considered statistically significant

CI, confidence interval; rhEPO, recombinant human erythropoietin

^*^p < 0.05, rhEPO group *vs.* control group using Fisher exact test;

^**^p < 0.01, comparing ROP outcomes with gestational age, birth weight using Fisher exact test

(Table 3).

For type 2 ROP, in infants with a gestational age of < 28 weeks, the increasing tendency in ROP was observed (12.5% vs. 6.0%, p = 0.342). The administration of rhEPO actually led to a reduction in type 2 ROP incidence in infants with a gestational age of 28–29^6/7^ weeks (1.1% vs*.* 4.9%, p = 0.002) and a birth weight of 1000–1499 g (1.2% vs*.* 4.2%, p = 0.002), as well as in infant boys (1.8% vs. 4.3%, p = 0.021) in comparison to that in the control group. Interaction analysis showed that sex, gestational age, or birth weight (p = 0.022, p = 0.016 and p = 0.013 respectively) may be the protective effect of rhEPO against type 2 ROP (Table 3).

For combined type 1 ROP or type 2 ROP, the administration of rhEPO significantly reduced the incidence of ROP (3.6% vs. 7.8%, p = 0.004) in infant boys, those with a gestational age of 30–32 weeks (2.9% vs. 6.2%, p = 0.014), and those with a birth weight 1,000–1,499 g (3.1% vs. 7.2%, p = 0.002). There were associations between rhEPO and sex, gestational age, or birth weight (p = 0.021, p = 0.012 and p = 0.008 respectively) (Table 3), which means sex, gestational age, and birth weight could have an influence on the protective effect of rhEPO against combined type 1 ROP or type 2 ROP.

Multivariate logistic regression analysis revealed that the administration of rhEPO led to a decrease in the incidence of type 2 ROP, in comparison to the control group. The coefficient was -0.646 (odds ratio, 0.524; 95% CI 0.284 to 0.965) (Table 4). Except for rhEPO treatment, the birth weight was also associated with the development of ROP, and greater birth weight was, therefore, protective factors for type 2 ROP (Table 4).

**Table 4:** Logistic regression analysis of risk factors for type 2 ROP

	B	S.E.	Wald	df	Exp(B) (95% CI)		P value
EPO	− 0.646	0.313	4.264	1	− 0.524 (0.284-0.965)		0.039
Birth weight	− 0.002	0.001	13.291	1	− 0.998 (0.997–0.999)		< 0.001
NEC	− 0.130	0.631	0.042	1	− 0.878 (0.258–3.02)		0.837
IVH	0.047	0.557	0.007	1	1.048 (0.352–3.121)		0.933
Constant	0.095	0.773	0.015	1	1.100		0.902

CI, confidence interval; SE, standard error; Wald, Wald test. rhEPO, recombinant human erythropoietin; NEC, necrotizing enterocolitis; IVH, intraventricular hemorrhage

### Safety analysis

At the baseline, before the administration of rhEPO, complete blood counts of the infants in the rhEPO group and those of the infants in the control group were similar. On day 14, after the last dose of rhEPO was administered, red blood cell count, haemoglobin, and haematocrit in infants in the rhEPO group [(3.3 ± 0.5) × 10^12^/L, 106.3 ± 23.6 g /L, and 32.2% ± 7.1%, respectively] were significantly higher than those of infants in the control group [(3.0 ± 0.5)× 10^12^/L, 98.2 ± 18.3 g/L, and 29.8 ± 5.5%, all p < 0.001, respectively]. Accordingly, transfusion requirements were much lower in the rhEPO group (1.5 ± 1.7) than in the control group: [1.8 ± 1.9, RR (95% CI) 0.302 (0.181–0.424), p < 0.001] (Table 5).

**Table 5:** Blood analysis before and after rhEPO administration

	Control n = 948	rhEPO n = 950	Difference (95% CI)	*P* value
Before rhEPO administration				
RBC count (× 10^12^/L),	4.0 ± 0.8	3.9 ± 0.6	0.06 (0.00–0.12)	0.065
Hemoglobin (g/l)	139.5 ± 29.1	140.8 ± 27.6	1.30 (− 1.25–3.85)	0.318
Hematocrit (%),	42.3 ± 8.8	42.7 ± 8.4	0.40 (− 0.37–1.17)	0.311
Platelet (10^9^/L	239.1 ± 114.2	246.1 ± 108.4	7.00 (− 3.02–17.02)	0.171
After rhEPO administration				
RBC count (10^12^/L)	3.0 ± 0.5	3.3 ± 0.5	0.30 (0.25–0.35)	* < 0.001*
Hemoglobin (g/l),	98.2 ± 18.3	106.3 ± 23.6	8.10 (6.20–10.00)	* < 0.001*
Hematocrit (%)	29.8 ± 5.5	32.2 ± 7.1	2.40 (1.83–2.97)	* < 0.001*
Platelet (10^9^/L)	265.3 ± 124.1	268.8 ± 109.7	3.50 (-7.04–14.04)	0.515
Transfusions	1.8 ± 1.9	1.5 ± 1.7	0.302 (0.181–0.424)	* < 0.001*

Overall, rhEPO was well tolerated in the current treatment protocol. In very preterm infants, there were no differences between the rhEPO and control groups in liver and renal functions or in electrolyte levels (data not shown). In addition, no adverse effects, including allergic reactions, venous thromboses, rashes, or seizures, were observed in either the rhEPO or the control group.

## Discussion

In these randomized trials reanalysis, we found that the effect of early administration of rhEPO on ROP in very preterm infants is associated with the gestation age, birth weight. Overall, low dose rhEPO treatment did not have an impact on the incidence of ROP development in very preterm infants. Rather, it was associated with a reduced risk of ROP in some groups of preterm infants. The incidence of type 2 ROP was reduced in infants with a gestational age between 28 and 30 weeks and bodyweight of 1000–1499 g compared to the control group. The administration of low dose rhEPO also reduced the incidence of type 2 ROP in infants with a gestational age between 28 and 29^6/7^ weeks and with a birth weight between 1000 and 1499 g.

To note, in infants with a gestational age of < 28 weeks and with a birth weight of < 1000 g, there was a non-significant increased incidence of ROP. This may indicate that the effect of EPO on ROP in preterm infants might be gestational age- and birth weight-dependent. In this subgroup of infants with a gestational age of < 28 weeks, we only have 76 infants in the rhEPO treatment group and 71 in the control group, and infants with birth weight < 1000 g, we only have 73 in the rhEPO treatment group and 74 in the control group. Considering the small number of ROP, the results from the current study for this group of infants is not conclusive. Indeed, in the most recently closed Preterm Erythropoietin Neuroprotection Trial (PENUT) for assessing the safety and efficacy of early high-dose erythropoietin for neuroprotection in extremely preterm infants, they found that in contrast to previous meta-analyses, treatment with rhEPO did not result in a higher rate or greater severity of retinopathy of prematurity than placebo [32].

In the subgroup analysis, rhEPO led to a decrease in the rate of type 2 ROP in infant boys but not in infant girls, suggesting a sex-differential response to rhEPO. It has been reported that the overall production of EPO is suppressed by oestrogen in rodents under normoxic or hypoxic conditions [33]. This finding suggests that sex hormones may modulate the non-haematopoietic EPO response. This also raises the possibility that EPO in infant boys is protective against ROP, whereas, in infant girls [34], against oestrogen-regulated EPO production in the reproductive organs [33]. This is consistent with findings from our study, which suggests that the benefit of EPO treatment on ROP is evident in infant boys but not in infant girls. Studies of the interaction between oestrogen and EPO signalling may further elucidate the direct and indirect contributions of oestrogen to sex-specific responses during EPO treatment, particularly in non-haematopoietic tissues [35].

Anaemia of prematurity is a common condition in preterm infants, particularly in extremely preterm infants, born before 28 weeks of gestation [6, 36]. Early rhEPO treatment reduces the numbers of red blood cell transfusions in low birth weight infants [37]. However, it is unclear whether rhEPO treatment is a risk factor for ROP [6]. In our subgroup analysis, there was a non-significant increase in the incidence of type 2 ROP in infants with a gestational age of < 28 weeks as well as in the incidence of type 1 ROP in infants with a birth weight of < 1000 g, following early rhEPO treatment. This indicates that there may be a gestational age- and birth weight-dependent effect of rhEPO on ROP. Anaemia during the first postnatal week remained an independent significant risk factor for ROP requiring treatment. Reducing early anaemia in preterm infants may reduce their risk of developing ROP [6, 38].

There were some limitations to the current study. First, EPO concentration was not measured longitudinally, even though this had been performed in our first rhEPO clinical studies (during which rhEPO was administrated to term infants with hypoxic ischemic encephalopathy) [39]. Therefore, we cannot establish the relationship between true EPO concentration and the rate of ROP in the current study to provide further information on the relationship between EPO serum concentration and ROP. Second, there were more male than female infants in the current study, which was a similar imbalance in sex ratio reported in other clinical studies with Chinese populations [40, 41]. In addition, this study was only hospitals of Zhengzhou University, indicating that the sample size was not large enough, especially for the infant groups that had a gestational age of < 28 weeks and a birth weight of < 1000 g. Therefore, results obtained in these infant groups need to be interpreted with caution, especially because these two groups of preterm infants showed a statistically non-significant increase in the incidence of ROP following rhEPO administration. Lastly, the data presented is retrieved from preterm infants enrolled in our previous clinical trials that none of them was originally designed for evaluation of the effect of rhEPO for ROP. Hence, a more appropriately designed, list ROP as a primary outcome, multi-centre studies with a larger sample are needed to further explore the impact of the administration of rhEPO on the incidence of ROP in very preterm infants.

## Conclusions

The observational analysis retrieved from our previous prospective randomised clinical trial data found that early repeated low-dose administration of rhEPO is safe for very preterm infants and reduced the need for red blood cell transfusions, but no significant effect on the overall incidence of ROP, even though the gestation age- and birth weight-dependent effect of rhEPO administration on ROP in preterm infants has been noticed. However, considering the overall relatively low incidence of ROP among preterm infants, data from more large-scale clinical studies are needed to derive more conclusive results regarding rhEPO administration in very preterm infants.

## Data Availability

The datasets used and/or analyzed during the current study are included in this published article.

## Abbreviations

ROP: Retinopathy of prematurity
rhEPO: Recombinant human erythropoietin
NICU: Neonatal intensive care units
PMA: Postmenstrual age
BPD: Bronchopulmonary dysplasia
NEC: Necrotizing enterocolitis
IVH: Intraventricular hemorrhage
